# The Therapy of Sciatica

**Published:** 1907-08

**Authors:** E. S. McKee

**Affiliations:** Cincinnati, O.


### PROGRESS IN MEDICAL SCIENCE.

### The Therapy of Sciatica.

By E. S. McKEE M.D., Cincinnati, O.

### COMPRESSES AND SCOTTISH DOUCHES.

DR. ERNST SOMMER, of Winterthur (*Wurtzburger Abhandlung ad. gesammten gebed pract. med. vi.* 4) The principal role of treatment reported was that played by compresses, Scottish douches, complete baths with movements, warm packs, massage. The author places the percentage of cures at from 80 to 90. The remarkable results attained by the treatment in the Brieger hydropathic institute in Berlin were described in particular. The work of the author demonstrated what we know about cause, treatment, and cure of sciatica.

### INJECTION TREATMENT OF SCIATICA.

LANGE (*Muenchner Med. Wooenschrift*), after trying in vain various medical and surgical means for relief in sciatica, finally found complete cure in five cases by the injection treatment. He injected a solution of B-eucaine, in the region of the sciatic notch, dissolved in 8 per cent. salt solution. When a large weal appeared under the skin, the needle was pushed down until a jerking showed that a nerve had been touched. Then 70 to 100 cc. were rapidly injected. Mild local pain lasted for two or three days. In three of his cases a second injection was required for complete cure. Functional and complete relief was almost instantaneous and quite permanent in all cases.

### CAUTERY AND GALVANIC TREATMENT—EXPLORATORY INCISION.

LISZNSKY (*Med. Record*) calls attention to the fact that superficial linear cauterisation with the Paquelin cautery along the course of the nerve trunk and over the sacral region is often remarkably efficacious in relieving the pain. The continuous galvanic current has also proven serviceable in the author's hands at the end of the first week. Mild cases may be treated by application of massage, hot cloths and rectal irrigation. Most of the acute cases under proper treatment recover promptly but with the probability of recurrence. Systematic preventive measures should be kept up for some months. Change of occupation is often necessary from the sedentary to the active. The sedentary individual should sit on a soft cushion or an air cushion to protect the nerve from pressure and injury. Those whose vocation necessitates violent muscular exercise of the extremities find the rest cure essential in many cases. Hydrotherapy, judiciously administered, should never be omitted. It has many cures to its credit. The wet pack applied at night is a very excellent means for relieving the pain as well as for influencing favorably the neurotic process. For this purpose we make use of the leg of a heavy pair of drawers which is dipped in water at 65 degrees F. and placed in position like a stocking. A roller bandage is then applied so that the leg can be kept in perspiration all night. This is removed in the morning and followed by a warm water ablution and massage. Ten or twelve packs result in much improvement. A certain percentage of patients prove unamenable to treatment when afflicted with the chronic form. This is usually due to their inability to pursue a persistent or systematic plan of treatment, or the physician, or more probably physicians who have had the case in charge, have not sufficiently studied the case. In long standing and permanent cases the author is in favor of exploratory incisions, for the purpose of exposing the nerve trunk, incising its sheath and freeing it from surrounding adhesions. Prognosis is better in the young than in the old, and in those of fair general health than in those of renal disease or diabetes. The more pronounced neurotic processes are not so rapidly amenable to treatment as the milder type and one attack predisposes to another.

STRYCHNINE INJECTIONS.

RETRIVOR (*Vratchebenaya Gazetta* 22, 1906,) reports nine cases in which the most favorable results followed the daily injections of 0.01-0.02 strychnine in the region of the painful part. While previously ordered remedies had proven worthless, all cases improved after the strychnine injections.

OSTWALD (*Berlin Klin Wochenschrift*) has found the deep injections of alcohol-cocaine and alcohol-stovaine in sciatica, trigeminal and other neuralgias to be very beneficial. He uses 80 percent. of alcohol to which is added 0.01 of cocaine or stovaine. He obtains relief in 90 percent. of the cases in from two to four injections. Relapses occurred in about one-third of the cases, but succumbed readily after one or two injections. These relapses occurred usually after the fourth or fifth month.

STERILISED AIR INJECTIONS.

THE treatment of sciatica and neuralgia by hypodermic injections of sterilised air has been tried not only at Lyons but also at Bordeaux and Paris. (*Journal de med. de Bordeaux.*) This journal has recently published thirteen cases (three sciatica and ten intercostal neuralgia) which had proven quite refractory to treatment, and had been cured by this means. All who have employed the technic indicated note that they have had no complications. The technic is very simple. After sterilising the region where the injection is to be made a sterilised hypodermic needle is inserted under the skin, and as soon as one is sure that no blood-vessel has been punctured, a rubber tube is joined onto the needle, and air from a rubber bag is injected from simple compression. To be quite safe it is best to place a little glass tube with cotton wadding between the needle and the bag. The injection should be stopped when the patient no longer complains of pain. A slight amount of massage should be carried out afterwards and repeated every day till crepitation has disappeared.

### SCIATICA SURGICALLY TREATED.

Pers, Copenhagen (*Deutch med. Wochenschrift*) reports two cases which were permanently cured by operation. The surgical treatment consists of laying the nerve free and making it clear from all adhesions. These were loosened and extirpated so that the nerve presented again a normal white appearance. Both patients complained only a short time of pain which, however, was less than before the operation. After about 30 days the patients were well and could leave the institution. Both after the lapse of two years have found themselves entirely free from pain. On account of these two typical cases Pers thinks that the surgical treatment of this trouble has a future.

### ELECTRICITY IN SCIATICA.

Juettner in his modern Physio-therapy says: "Pressure, compression bandage, massage and vibration. Dry heat. Rest limb in elevated position. Galvanism as before. Static spray (positive) locally. Local sun baths. The local treatment of any neuralgia must always be secondary to the general treatment of the system. Local treatment is desirable, general treatment is necessary. Even in old obdurate cases, good results can be achieved if the physician will persist in handling his patient generally and locally according to the directions and suggestions given above. The apparent anodyne action of the faradic current in neuralgic cases (i.e. sciatica), is due to its alterant action on the muscular tissue and through the latter on the circulation. The blood supply is regenerated and the cry of the nerve for healthy blood is stilled. The use of violent faradic shocks is never indicated except in paralytic conditions when it is a question of making an impression on the tissue that has hardly any vitality left in it. Painful applications of the faradic current are never proper."

### BATH AND DRUG TREATMENT.

Harburn (*Lancet, Feb.* 4, '05,) has found no combination of drugs in the acute stage to equal the following: Aspirin gr. 6 or 0.36, phenacetin gr. v. or 0.33, quinine salicylate gr. 2 or 0.12 and codeine gr. ¼ to ½ or 0.015 to 0.03. First clean out the bowels with calomel followed by salines. In the subacute stage nothing is more serviceable than the half combined bath. Patients sits in a vapor bath which comes up to the waist line only. This while it does not exhaust the patient as much as the full bath of vapor allows a higher temperature to be borne by the affected part. 110 degrees F. can be tolerated for from 10 to 15 minutes. At the end of this time the patient sits in a bath of the Buxton mineral water, heated to a temperature of 95 degrees F. for eight minutes, and during the last three minutes a hot undercurrent douche at 102 degrees F. to 110 F. is applied to the affected limb. Chronic form. Where neuritis is not present the Aix massage baths with the douche applied to the painful parts, are of great value as are also dry and electric massage followed by gentle stretching of the nerve in cases where adhesions are present. The Buxton swimming bath at a temperature of 82 degrees F. is one of the most valuable means of treatment at our disposal. In true neuritis, however, massage is, as a rule, not beneficial, and nerve stretching is contraindicated. The combined bath, alternating with the natural swimming bath and the application of electricity in the form of the constant current (5 to 15 milliamperes), ascending or descending over the affected nerve or in the form of the constant current bath, are of much service. The affected limb should be kept warm by wearing double legged pants of wool. The hypodermic injection of pilocarpine nitrate on alternate days for two or three weeks, except when there is organic heart disease is highly praised.

Shoemaker (*Virginia Med. Semi-Monthly*) recommends deep injections in the region of the nerve of atropine sulphate 1-150 gr. three times daily, also injections of cocaine as near the nerve as possible. Baking the limb once daily is one of the best means of assisting in the cure of the disease. This should be done in a heating apparatus. The limb is wrapped up in a blanket and placed in an apparatus, and the temperature is gradually run up to 300 to 400 degrees F., and kept there for at least an hour. When gout or rheumatism is the cause, treat that first, and often the sciatica needs no special treatment. The same is true when the disease is due to other causes. Pathologically speaking, traumatic sciatica is perineuritis. The nerve looks red and inflamed from hyperemia of the vasonervorum. Sciatica, especially in women, is often due to pelvic tumors: hence a vaginal and rectal examination should be made in intractable cases in women.

### EN RESUME.

The first essential to the successful treatment of sciatica, the hip gout of Pliny, is a thorough knowledge of the individual patient in hand. We should in the beginning, institute a most exhaustive physical examination not only of the sciatica nerve, but also of the entire nervous system and the patient's whole body, family history, diseases, mode of living, place of living, business and habits of life and diet. If the patient is a woman, especial attention should be given to a careful rectal and vaginal examination, for the disease in woman is often due to pelvic tumors. One can not know too much about his patient, suffering from this obscure malady, to assist him in the cure. Constitutional elimination and general therapeutic measures to relieve pain and promote sleep should first follow that best of all starters, a mercurial purge followed by a saline, which treatment should be instituted as soon as the diagnosis is positively settled and the causal relations made clear. Morphia should be used with extreme caution, owing to the danger of forming the habit. Rheumatic cases are usually benefited by the salicylates, syphilitic cases by the iodides, gouty cases by colchicum and the salines. One of the best combinations of drugs in the acute stage is the following:

℞-Aspirin gr. vi or 0.36.
Phenacetine gr. v or 0.33.
Quiniæ salicylate gr. ii or 0.12.
Codeinæ sulphatis gr. ¼ to ½ or 0.015 to 0.03.

Having first cleaned out the bowels with calomel and salines, this, in capsule should be repeated every two or three hours.

*Injection Treatment.*—Hypodermics of very large doses of strychnine in the region of the painful part, has cured cases which were rebellious to every other plan of treatment. Injections into the region of the nerve of atropine sulphate 1-150 of a grain three times daily, also cocaine injections as near the nerve as possible are frequently followed by success. Deep injections of alcohol-cocaine and alcohol-stovaine, 80 per cent. alcohol and the incorporation of 0.01 of cocaine or stovaine. Relief is obtained in about 90 per cent. of cases in from two to four injections. Relapses, generally after the fourth or fifth month, occur in about one-third of the cases but yield readily after one or two injections. Beta encaine, 6%, in 8 per cent. salt solution should be injected in the region of the sciatic notch. When a large weal appears under the skin, the needle is pushed down till a jerking shows that a nerve had been touched, then 70 to 100 cc. are rapidly injected. Functional and complete relief is almost instantaneous. In a portion of the cases only is a second injection necessary for complete cure. The hypodermic injection of sterilised air is conducted as follows: After sterilising the region where the injection is to be made, a sterilised hypodermic needle is inserted under the skin, and as soon as one is sure that no bloodvessel has been punctured, a rubber tube is joined onto the needle and air from a rubber bag is injected by means of simple compression. To be quite safe, it is well to place a glass tube with a little cotton in it, between the needle and the bag. The injection should be stopped when the patient no longer complains of pain. A slight amount of massage should be used every day till crepitation disappears.

The *rest cure* of Weir Mitchell is beneficial in some cases, and the fixation of the limb in plaster of Paris is good treatment, especially in those cases where the vocation necessitates violent exercise of the lower extremities. Change of occupation is often necessary from the sedentary to the active or vice versa. The sedentary person should sit on a soft cushion or an air cushion, to protect the nerve from pressure or injury.

*Hydrotherapy* judiciously administered should always be given consideration. It has many cures to its credit. The wet pack administered at night is a very excellent means of relieving pain as well as for influencing favorably the neurotic process. For this purpose we may use the leg of a heavy pair of drawers dipped in water at 65 degrees F., and placed in position like a stocking. A roller bandage is then applied so that the leg may be kept in perspiration all night. This is removed in the morning and followed by a warm water ablution and massage. Ten or twelve packs usually result in much improvement. The half combined bath in the subacute stage proves quite serviceable. Patient sits in a vapor bath which comes up to the waist line only. This, while it does not exhaust the patient as much as the full bath of vapor, allows a much higher temperature to be born by the affected part. 110 degrees can be tolerated for from 10 to 15 minutes. At the end of this time, the patient sits in a bath heated to a temperature of 95 degrees F. for eight minutes, and during the last three minutes a hot undercurrent douche, at 102 to 112 degrees F., is applied to the affected limb. The combined bath alternated with the natural swimming bath is of value. The internal bath by the ingestion of large quantities of water is well advised.

*Electricity*.—Static spray (positive) locally. The galvanic current should be applied to the nerve from four to eight minutes, and should not exceed from three to five milliamperes. When the nerve substance has been involved, gentle muscular stimulation with the uninterrupted galvanic current keeps the structures in good nutrition and prevents atrophy. Faradism. The apparent anodyne action of this current in sciatica is due to its alterant action on the muscular tissue, and through the latter on the circulation. The blood supply is regenerated and the cry of the nerve for healthy blood is stilled. Painful applications of the Faradic current are not proper.

*Surgical Treatment.*—In cases of long standing it is advisable to make an exploratory incision for the purpose of exposing the nerve trunk, incising its sheath and freeing it from surrounding adhesions. Some good results of nerve stretching are reported and many bad. It is an operation which has not gained much commendation from the general medical mind. Myelitis has in a few cases followed this operation, and nerve stretching is contraindicated where neuritis is present. There is a substitute operation called bloodless nerve stretching, in which the patient under ether, the thigh is forcibly flexed upon the pelvis and the leg extended at the knee and this position maintained for some minutes.

Massage along the course of the nerve, even though painful, is often of benefit by relieving the nerve of adhesions. In true neuritis massage is as a rule not beneficial. Massage, or what is better, mechanical vibration is of value in the chronic stage where atrophy has commenced.

Cure is easier in the young than in the old, and in those of fair general health than in those suffering from the various serious chronic diseases. The more pronounced neurotic processes are not so amenable to treatment as the milder types, and one attack predisposes to another. The reason that some patients do not recover, is that they are unable to pursue a persistent or systematic plan of treatment, and the physician, or more probably the physicians, who have had the case in hand have not had opportunity, owing to the frequent changes, to sufficiently study the case. Otherwise the failure to cure must be due to the medical man not having studied his patient thoroughly enough, having overlooked some point. The only thing for him to do is to commence at the beginning and go it all over and try to ascertain wherein he has failed, for he has failed somewhere. An exact diagnosis of the conditions is one of the first and last means of cure.

